# Analyses of gene expression profiles in the rat dorsal horn of the spinal cord using RNA sequencing in chronic constriction injury rats

**DOI:** 10.1186/s12974-018-1316-0

**Published:** 2018-09-25

**Authors:** Hui Du, Juan Shi, Ming Wang, Shuhong An, Xingjing Guo, Zhaojin Wang

**Affiliations:** 1 Department of Histology and Embryology, Taishan Medical University, Taian, 271000 China; 2 Department of Human Anatomy, Taishan Medical University, Taian, 271000 China; 3 Department of Physiology, Taishan Medical University, Taian, 271000 China

**Keywords:** Dorsal horn, Chronic constriction injury, RNA sequencing, Differentially expressed genes

## Abstract

**Background:**

Neuropathic pain is caused by damage to the nervous system, resulting in aberrant pain, which is associated with gene expression changes in the sensory pathway. However, the molecular mechanisms are not fully understood.

**Methods:**

Wistar rats were employed for the establishment of the chronic constriction injury (CCI) models. Using the Illumina HiSeq 4000 platform, we examined differentially expressed genes (DEGs) in the rat dorsal horn by RNA sequencing (RNA-seq) between CCI and control groups. Then, enrichment analyses were performed for these DEGs using Gene Ontology (GO) function, Kyoto Encyclopedia of Genes and Genomes (KEGG) pathway, Hierarchical Cluster, and protein-protein interaction (PPI) network.

**Results:**

A total of 63 DEGs were found significantly changed with 56 upregulated (e.g., Cxcl13, C1qc, Fcgr3a) and 7 downregulated (e.g., Dusp1) at 14 days after CCI. Quantitative reverse-transcribed PCR (qRT-PCR) verified changes in 13 randomly selected DEGs. GO and KEGG biological pathway analyses showed that the upregulated DEGs were mostly enriched in immune response-related biological processes, as well as 14 immune- and inflammation-related pathways. The downregulated DEGs were enriched in inactivation of mitogen-activated protein kinase (MAPK) activity. PPI network analysis showed that Cd68, C1qc, C1qa, Laptm5, and Fcgr3a were crucial nodes with high connectivity degrees. Most of these genes which have previously been linked to immune and inflammation-related pathways have not been reported in neuropathic pain (e.g., Laptm5, Fcgr3a).

**Conclusions:**

Our results revealed that immune and defense pathways may contribute to the generation of neuropathic pain after CCI. These mRNAs may represent new therapeutic targets for the treatment of neuropathic pain.

**Electronic supplementary material:**

The online version of this article (10.1186/s12974-018-1316-0) contains supplementary material, which is available to authorized users.

## Background

Neuropathic pain is a chronic pain and may result from primary damage, disease or dysfunction of the peripheral or central nervous system, which is characterized by an increased responsiveness of nociceptive neurons in the nervous system [1]. The molecular mechanisms of neuropathic pain remain poorly understood, but it is known to involve nerve injury, inflammatory cytokine release, anatomical remodeling, and nociceptive receptors. [2, 3]. Thus, an improved understanding of pathogenesis from gene interactions in neuropathic pain is crucial for the development of the genetic and various neurobiological base therapeutic strategies to prevent neuropathic pain and improve the treatment effect.

The chronic constriction injury (CCI) rat model which simulates the symptoms of chronic nerve compression has been used as a model of neuropathic pain because rats subjected to CCI behave analogously to humans with neuropathic pain [4, 5]. Reportedly, CCI is highly associated with inflammation [6, 7]. Activation of immune and immune-like glial cells in the injured nerve, dorsal root ganglia, and spinal cord could generate a variety of mediators such as cytokines, chemokines, and other inflammatory mediators [8]. Interestingly, some of these mediators, such as cytokines and chemokines, can directly activate or sensitize nociceptors, contributing to the development of neuropathic pain [9].

Gene expression profile studies can be used to provide understanding of the molecular mechanisms underlying the development and maintenance of neuropathic pain [10–12]. Some studies using microarray and RNA sequencing (RNA-seq) analysis have been conducted to investigate the mechanism underlying the generation of neuropathic pain in spared nerve injury (SNI) model [13, 14]. Although they identified several crucial differentially expressed genes (DEGs) and different immune actions in SNI models, the alteration of gene expression and mechanisms on neuropathic pain are still unclear. Therefore, the present study was carried out to compare the different gene expression profiles of the dorsal horn of CCI rats and controls using the Illumina Hiseq 4000 to reveal the underlying regulatory mechanism of CCI rat models. Moreover, the molecular and cellular functions of the predicted mRNAs as well as the signaling pathways involved based on the present experiment will be further investigated.

## Methods

### Animals

Adult male Wistar rats weighing 200–250 g were obtained from the Animal Center of Taishan Medical University. All experimental procedures followed the guidelines of the Taishan Medical University Institutional Animal Care and Use Committee. Efforts were made to minimize the number of animals used and their sufferings.

### CCI models

CCI to the sciatic nerve of the right hind limb in rats was performed based on previous description [15]. Briefly, animals were anesthetized with 4% chloral hydrate (10 ml/kg; i.p.). The sciatic nerve of the right hind limb was exposed at the middle of the thigh by blunt dissection. To prevent the interruption of blood circulation through the epineural vasculature, four chromic gut ligatures were loosely tied (4.0 silk) around the nerve with spacing at ~ 1 mm. In the control group, the right sciatic nerve was exposed for 2–3 min, but was not ligated. Following surgery, the skin was closed with a single suture, and the animals were allowed to recover for various period of time before behavioral testing.

### Mechanical withdrawal threshold (MWT)

All behavioral tests were performed in a blinded manner. Mechanical allodynia and thermal hyperalgesia are reproducible and sensitive behavioral readouts of neuropathic pain. Behavioral testing was conducted prior to surgery and on days 1, 3, 7, 10, and 14 following surgery. Animals were allowed to acclimate to elevated cages (20 × 14 × 16 cm) with a wire mesh bottom. MWT was measured by assessing hind paw sensitivity to innocuous mechanical stimulation. Von Frey filaments (0.41–15.1 g; North Coast, Gilroy, CA) were applied to the plantar aspect of the right hind paw. Lifting, licking the paw, and running away were all considered as positive responses. The maximum applied pressure was recorded. The MWT of each animal was the average of six measurements taken at 5 min intervals.

### Thermal withdrawal latency (TWL)

In this assay, rats were placed in a transparent, square, bottomless acrylic box (17 × 11.5 × 14 cm) and allowed to adapt for 20 min. Responses to thermal stimulation were evaluated using a 37,370 plantar test apparatus as a source of radiant heat. A beam of focused light set at 60 °C was directed towards the plantar surface of the hind paw, and the maximum latency time was recorded. The time to purposeful withdrawal of the foot from the beam of light was measured. A cutoff time was set at 40 s to prevent tissue damage. Every hind paw was tested alternately at 5 min intervals. The results obtained for each rat were expressed in second as the mean of six withdrawal latencies. Finally, the average value was used for statistical analysis.

### Tissue collection, RNA isolation, cDNA library preparation, and sequencing

Animals were deeply anesthetized with isoflurane (3%) at 14 days after surgery and perfused through the ascending aorta with normal saline (100 ml, 4 °C). The L4–5 spinal cord segments that correspond to L4–5 spinal nerve roots and match L1 vertebral level were dissected. The dorsal horns of L4–5 spinal cord were collected. Total RNA was extracted from the dorsal horn tissue using Trizol reagent (Invitrogen, Carlsbad) according to the manufacturer’s protocol. RNA quantity and quality were measured using a NanoDrop ND-1000. The cDNA library was constructed using KAPA Stranded RNA-Seq Library Preparation Kit (Illumina) following the manufacturer’s protocol. Briefly, poly-(A) mRNA was isolated from total RNA using the NEBNext Oligo d(T) magnetic beads. Under an elevated temperature, mRNA was fragmented into small pieces after the fragmentation buffer was added. Using the mRNA fragments as templates, the first-strand cDNA was synthesized with random primers. Then, the second-strand cDNA was obtained using DNA polymerase I and RNase H. The synthetic cDNAs were end-repaired by polymerase and ligated with “A-tailing” base adaptors. Suitable fragments were selected for PCR amplification to construct the final cDNA library. The final double-stranded cDNA samples were verified with an Agilent 2100 Bioanalyzer (Agilent Technologies). After cluster generation (TruSeq SR Cluster Kit v3-cBot-HS, Illumina), sequencing was performed on an Illumina HiSeq 4000 sequencing platform.

### RNA-seq data processing

Image analysis, base calling, and error estimation were performed using Illumina/Solexa Pipeline version 1.8 (Off-Line Base Caller software, version 1.8). Quality control was checked on the raw sequence data using FastQC (https://en.wikipedia.org/wiki/FASTQ_format). Raw data were pre-processed using Solexa CHASTITY and Cutadapt to remove adaptor sequences, ribosomal RNA, and other contaminants that may interfere with clustering and assembly. The trimmed reads are mapped to the corresponding reference genome using HISAT2 (version 2.0.4) for RNA-seq, and StringTie (version 1.2.3) was used to reconstruct the transcriptome [16, 17]. Then, Ballgown software was applied to calculate the fragments per kilobase of exon per million fragments mapped (FPKM) in RNA-seq data and analyze DEGs [18, 19], with the FPKM ≥ 0.5 (Cuffquant) considered statistically significant.

### Bioinformatics analysis

The Gene Ontology (GO) functional and Kyoto Encyclopedia of Genes and Genomes (KEGG) pathway enrichment analysis were performed for DEGs using the Database for Annotation, Visualization and Integrated Discovery (DAVID) and KEGG Orthology-Based Annotation System (KOBAS) online tools (http://www.geneontology.org and http://www.genome.jp/kegg). Hierarchical cluster analysis was performed for enriched genes by Cluster 3.0 software. The protein-protein interaction (PPI) network of the proteins encoded by the DEGs was searched using STRING online software (http://string-db.org/).

### Quantitative reverse transcription-PCR (qRT-PCR) analysis

Thirteen DEGs (11 regulated and 2 downregulated genes) were randomly selected and detected by qRT-PCR. The expression of β-actin mRNA was also determined as an internal control. Total RNA was extracted from the dorsal horn tissue using Trizol reagent (Invitrogen) according to the manufacturer’s protocol. RNA concentration was determined spectrophotometrically. cDNA was synthesized using a cDNA synthesis kit (Invitrogen) according to the manufacturer’s instructions. Primer sequences are listed in the Table 1. qRT-PCR was performed in triplicates by using a 7300 real-time PCR system (Applied Biosystems, Foster City, CA) according to the manufacturer’s instructions. A comparative cycle of threshold fluorescence (∆Ct) method was used, and the relative transcript amount of target gene was normalized to that of β-actin using the 2^−∆∆Ct^ method. The final results of qRT-PCR were expressed as the ratio of test mRNA to control.

**Table 1:** The primers used in real-time PCR

Primers	Forward	Reverse	Amplicon size (bp)
Cxcl13	GGCCACGGTATTCTGGAGAC	CCATCTGGCAGTAGGATTCACA	192
Reg3b	GTCCTGGATGCTGCTCTCCT	GGCAACTAATGCGTGCAGAG	92
Plac8	AGGCAGCAACAGTTATCGTGAC	CTCATCGCCACCGTTGTTCC	196
C1qc	GTCAAGTTCAATTCCGCCATCAC	TGTGGTGGACGAAGTAGTAGAGG	103
Ccl2	CCTGCTGCTACTCATTCACTGG	TTCTGATCTCACTTGGTTCTGGTC	197
C1qa	TGTCTGTCTATCGTGTCCTCCTC	GATGCTGTCGGCTTCAGTACC	192
C3	TGTGAGCCTGGAGTGGACTAC	CTGAGCCTGACTTGATGACCTG	112
C1qb	AGGTGGCTCTGGAGACTACAAG	GAACTGGCGTGGTAGGTGAAG	198
C4a	CCAGACTCACATCTCCATCTCAAG	CCTCCAGGTCTCCGATCTCAG	80
Ngfr	CCTGCCTGGACAGTGTTACATTCTC	CAGTCTCCTCGTCCTGGTAGTAGC	132
Aif1	CCAACAGGAAGAGAGGTTGGA	CAGCATTCGCTTCAAGGACA	169
Urgcp	ACGTCAGCAGCAACTCCAAG	GGATTCGTGCCTAAGTTGAGGT	106
Dusp1	AGATATGCTCGACGCCTTGG	TGTCTGCCTTGTGGTTGTCC	122
β-actin	TGTCACCAACTGGGACGATA	GGGGTGTTGAAGGTCTCAAA	165

### Statistical analysis

Data are presented as the means ± SEM. The results from the behavioral study were statistically analyzed using repeated measures analysis of variance. qRT-PCR results were analyzed using one-way analysis of variance (ANOVA) followed by Tukey’s multiple comparison test. Significance was set at *p* < 0.05.

## Results

### Model identification of neuropathic pain

**Fig. 1:**
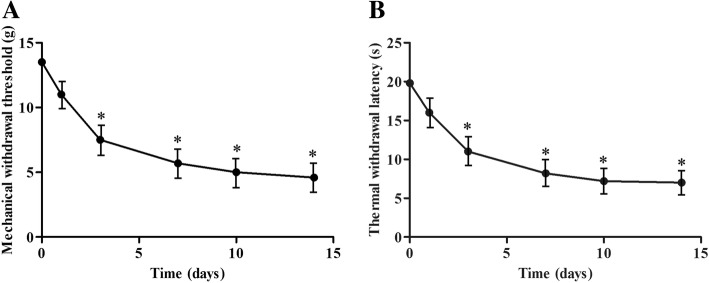
Nociceptive behavior developed in chronic constriction injury (CCI) model rats. Mechanical withdrawal threshold (MWT) in each time point (**a**) and thermal withdrawal latency (TWL) in each time point (**b**). *n* = 6, *p < 0.05 compared with controls

The neuropathic pain rat model was established by CCI to the sciatic nerve of the right hind limb in rats. Both mechanical allodynia and thermal sensitivity were determined in all CCI model rats at 0, 1, 3, 7, and 14 days after surgery, respectively. CCI rats exhibited higher sensitivity to mechanical and thermal stimuli from days 1 to 14. Both MWT and TWL reached a steady peak at day 14 after surgery (Fig. 1).

### Differential gene expression in the spinal cord

**Fig. 2:**
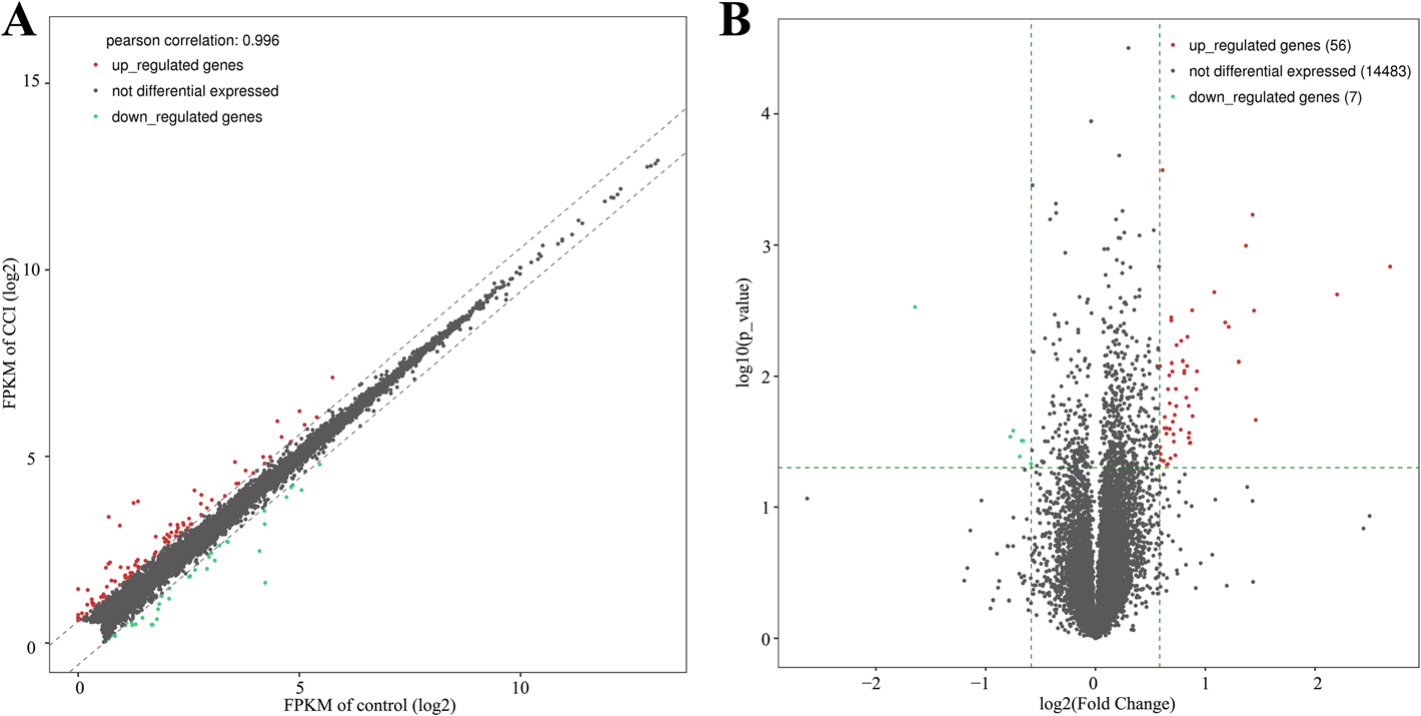
The differential expression of genes (DEGs) in the dorsal horn between control and chronic constriction injury (CCI) model was determined by RNA-seq technology. **a** Scatter plot showing the upregulated and downregulated genes (the red and green dots, respectively) in the dorsal horn of L4–5 spinal cord in the CCI rat with respect to the control. Black dots represent genes with no significant difference. **b** Volcano plot indicated upregulated and downregulated DEGs in the dorsal horn of CCI models. Red dots represent genes with significantly upregulated expression, green dots represent genes with significantly downregulated expression, while black dots represent genes with no significantly difference, respectively

To determine genes that are involved in the pathological process of neuropathic pain, the dorsal horn of L4–5 spinal cord of rats was analyzed using an Illumina HiSeq 4000 sequencing technique at 14 days after CCI surgery. Using the FPKM of ≥ 0.5, abundant expression levels were compared to those of CCI-induced neuropathic pain with control. We identified a total of 17,912 mRNA transcripts corresponding to 14,546 genes in CCI-induced neuropathic pain rat models after 14 days (Additional file 1: Table S1; Additional file 2: Table S2). Sixty-three genes were differentially expressed between CCI-induced neuropathic pain and control tissues (Table 2; Additional file 3: Table S3) using two criteria: a greater than 1.5 fold expression level change and *p* value ≤ 0.05 from ANOVA test. The related gene expression frequency and abundance in the dorsal horn of the CCI rat were showed in Fig. 2a. These 63 genes included 56 upregulated genes (e.g., Cxcl13, C1qc, Cgr3a) and 7 downregulated genes (e.g., Urgcp, Usp1) as shown in the volcano plot (Fig. 2b).

**Table 2:** The upregulated and downregulated genes in rat neuropathic pain model

Gene name	Locus	Fold change	*p* value	Biological process
Cxcl13	chr14:15253125-15258207	6.426350091	0.001461007	Chemokine-mediated signaling pathway
Reg3b	chr4:109467272-109470510	4.596165659	0.00238738	Negative regulation of cell death
Plac8	chr14:10692764-10714524	2.75098502	0.02157393	Negative regulation of apoptotic process
C1qc	chr5:155255005-155258392	2.72105301	0.003170161	Innate immune response
Ccl2	chr10:69412017-69413870	2.696533508	0.000586007	Glial cell migration
C1qa	chr5:155261250-155264143	2.585336373	0.001012602	Innate immune response
C3	chr9:9721105-9747167	2.470300657	0.007779142	Complement activation
C1qb	chr5:155246447-155252003	2.318601492	0.004223697	Innate immune response
C4a	chr20:4302347-4508214	2.268474076	0.003906162	Complement activation
C4a	chr20:2651599-2678141	2.117474349	0.002292613	Complement activation
Fcer1g	chr13:89601896-89606326	1.897610848	0.009152089	Innate immune response
Ngfr	chr10:83389847-83408061	1.889723015	0.012534711	Sensory perception of pain
Fcgr3a	chr13:89385859-89396051	1.846156689	0.020151454	Regulation of sensory perception of pain
Fyb	chr2:55835151-55983804	1.841013316	0.003152222	Immune response
Fcgr1a	chr2:198430530-198458041	1.820562407	0.032185553	Regulation of immune response
LOC103691423	chr2:23260651-23260965	1.812907143	0.032394733	
Cd22	chr1:89314558-89329418	1.806446581	0.027087834	Cell adhesion
Gapt	chr2:41869556-41871858	1.801373741	0.01683096	Innate immune response
Ly86	chr17:28104589-28191436	1.801122811	0.029526635	Innate immune response
Cd33	chr1:98398660-98402968	1.787375322	0.005028128	Regulation of immune response
Pld4	chr6:137323713-137331231	1.78323674	0.008341961	Phagocytosis
Ltc4s	chr10:35737664-35739625	1.774305996	0.014581973	Response to axon injury
Cd53	chr2:209489279-209537087	1.752658145	0.009085537	Cell surface receptor signaling pathway
Ctsz	chr3:172527107-172537877	1.750656844	0.009448762	Regulation of neuron death
Clec4a1	chr4:155947453-155959993	1.73513319	0.007661068	Adaptive immune response
Rpe65	chr2:266141581-266169197	1.717980882	0.005397693	Cellular response to electrical stimulus
Irf8	chr19:54314865-54336640	1.713827049	0.025622097	Phagocytosis
Atf3	chr13:109817728-109849632	1.667935639	0.005824841	Positive regulation of cell proliferation
Apobec1	chr4:155386711-155401480	1.665506432	0.016894935	Regulation of cell proliferation
Tmem176a	chr4:78458625-78462423	1.661562833	0.01247483	Cell differentiation
Cyp4b1	chr5:134508730-134526089	1.656053246	0.040180738	Cell differentiation
Gpr31	chr1:53519829-53520788	1.649462641	0.019751159	signal transduction
Aoah	chr17:45872414-46115004	1.639719248	0.031572447	Inflammatory response
LOC102557117	chr5:187312-187688	1.636194027	0.027745501	
Clec7a	chr4:163216163-163227334	1.629244213	0.022249116	Innate immune response
Bin2	chr7:142273833-142300382	1.625336862	0.00919119	Cell chemotaxis
Gpr34	chrX:10023489-10031167	1.618400204	0.007940879	Signal transduction
Mx1	chr11:37891156-37914983	1.613580093	0.003770887	Innate immune response
Gpr183	chr15:108364701-108376221	1.613459608	0.003571085	Adaptive immune response
AABR07001573.2	chr1:53220397-53284319	1.605472328	0.042391557	
Cd68	chr10:56268720-56270640	1.59862216	0.025231623	Neutrophil degranulation
AC115371.1	chr15:33606124-33611579	1.597574602	0.016091626	
Oosp1	chr1:228032983-228053645	1.593938444	0.009835678	Response to stimulus
Tmem176b	chr4:78450724-78458179	1.581317379	0.012542608	Cell differentiation
Adgre1	chr9:9431860-9585865	1.578724864	0.04691516	Adaptive immune response
Fcgr2b	chr13:89327794-89433815	1.569218908	0.024948119	Immune response
Cyth4	chr7:119820537-119845003	1.56917098	0.047508716	Regulation of molecular function
Aif1	chr20:5161333-5166448	1.565044457	0.027459054	Response to axon injury
Plek	chr14:100151210-100217913	1.548834783	0.020475153	Integrin-mediated signaling pathway
Wipf3	chr4:84597323-84676775	1.545203106	0.024900292	Cell differentiation
Itgad	chr1:199495298-199623960	1.537780288	0.044440601	Microglial cell activation
RGD1309350	chr1:213577122-213580542	1.527740296	0.000267242	Purine nucleobase metabolic process
Anxa3	chr14:14364008-14426437	1.51105029	0.043685994	Phagocytosis
Laptm5	chr5:149047681-149069719	1.508262862	0.03919455	Transport
Tmem154	chr2:183674522-183711812	1.504875551	0.034809815	
Csf1r	chr18:56414488-56458300	1.503532191	0.008357977	Cytokine-mediated signaling pathway
Urgcp	chr14:85957716-85991211	0.32013414	0.002977579	Cell cycle
LOC500300	chr4:148782479-148784562	0.58414901	0.0290017	Regulation of autophagy
Klhdc7a	chr5:158436757-158439078	0.594907041	0.026052604	Protein ubiquitination
AABR07026893.1	chr17:3729421-3729810	0.620034111	0.041049018	
Dusp1	chr10:16970626-16973418	0.627386061	0.031077843	Inactivation of MAPK activity
Plac9	chr16:3851270-3866008	0.633314755	0.03114494	
AABR07042903.1	chr19:14345993-14346891	0.666129407	0.046588447

### GO functional analysis of DEGs

**Fig. 3:**
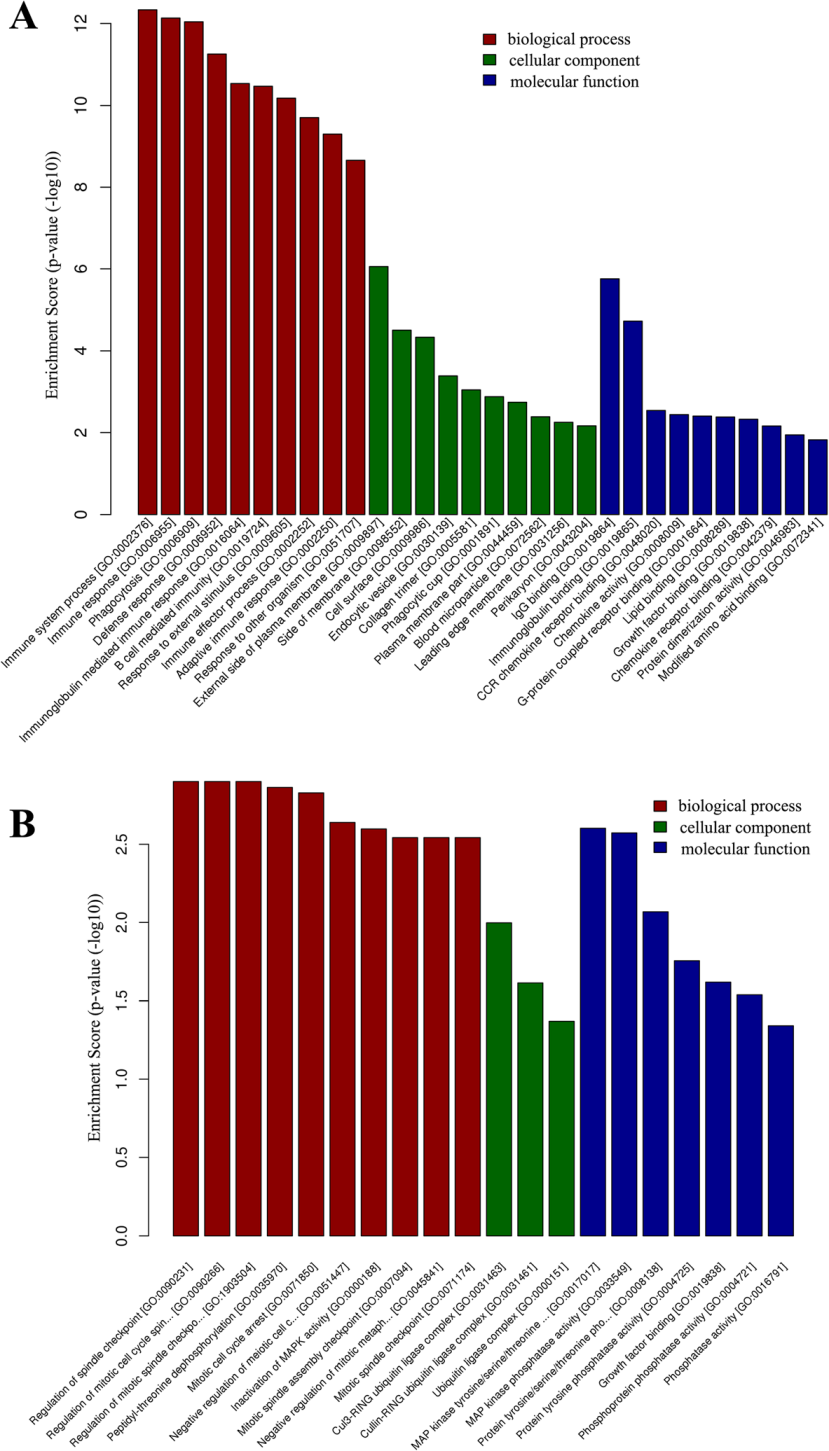
The Gene Ontology (GO) analysis of differentially expressed genes (DEGs) in the dorsal horn of chronic constriction injury (CCI) rats. GO annotation of upregulated DEGs (**a**) and downregulated DEGs (**b**) of CCI model versus control. Bar plots show the top ten enrichment score (−log_10_(*p* value)) values of the significant enrichment terms of DEGs involving biological process, cellular component, and molecular function

According to the functional annotation in GO database, the upregulated DEGs were mostly enriched in biological processes (BP) related to immune and defense responses (Additional file 4: Table S4), cellular component (CC) terms such as endocytic vesicle and phagocytic cup (Additional file 5: Table S5), and molecular function (MF) terms related to IgG binding and chemokine activity (Additional file 6: Table S6). The GO enrichment terms of BP, CC, and MF for upregulated DEGs are shown in Fig. 3a.

Meanwhile, the downregulated DEGs were enriched in BP terms such as regulation of spindle checkpoint and inactivation of mitogen-activated protein (MAP) kinase (MAPK) activity (Additional file 7: Table S7), CC terms such as Cul3-RING ubiquitin ligase complex (Additional file 8: Table S8), and MF terms such as MAP kinase phosphatase (MKP) kinase activity (Additional file 9: Table S9). The GO enrichment terms of BP, CC, and MF for downregulated DEGs are shown in Fig. 3b.

### KEGG pathway enrichment analysis of DEGs

**Fig. 4:**
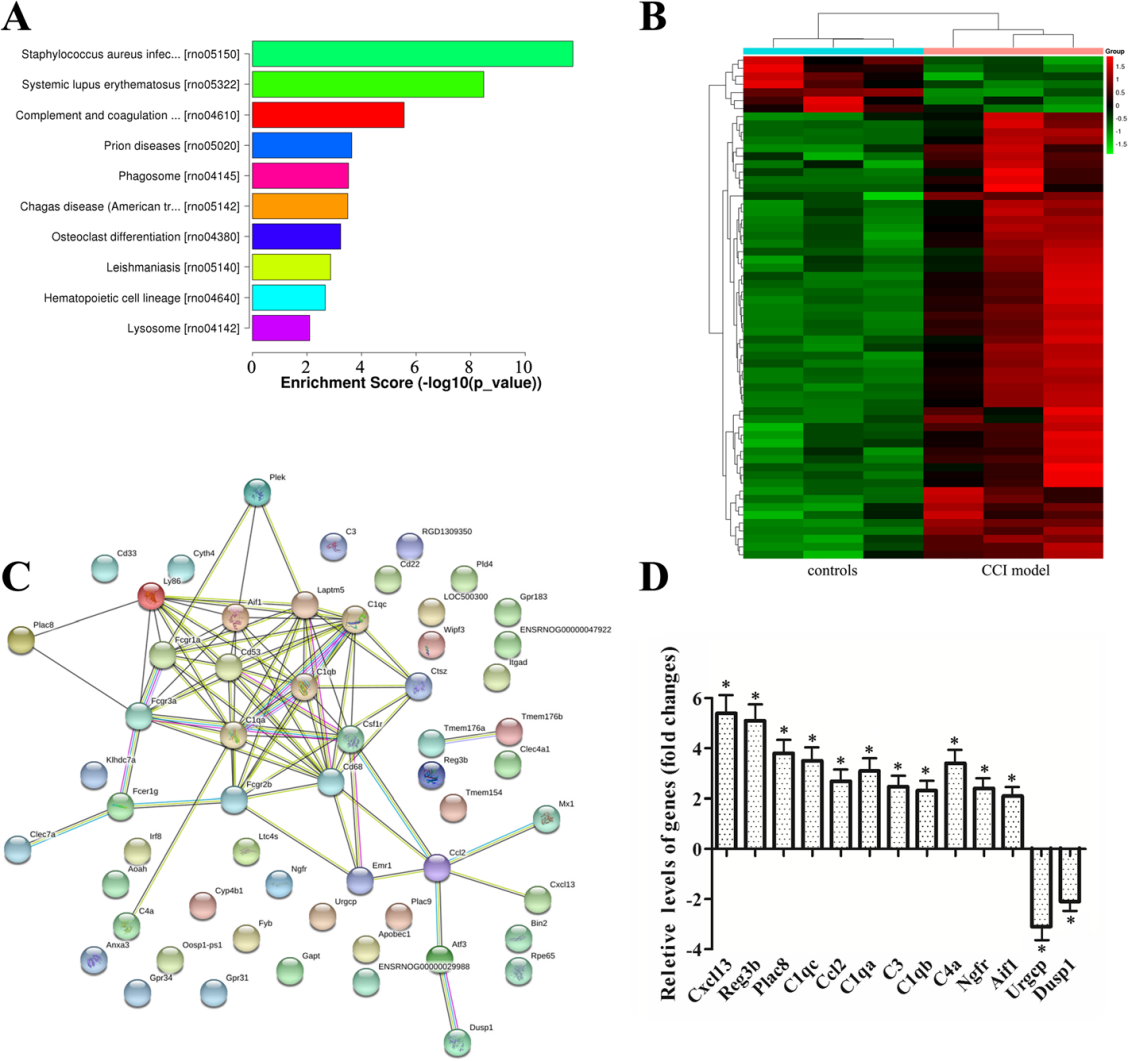
Kyoto Encyclopedia of Genes and Genomes (KEGG), hierarchical clustering, and protein-protein interaction (PPI) network analysis of differentially expressed genes (DEGs) in the dorsal horn of chronic constriction injury (CCI) rats. **a** Histogram of KEGG pathway enrichment distribution of DEGs. The bar plot shows the top ten enrichment score (−log_10_(*p* value)) value of the significant enrichment pathway. **b** Heat map of DEGs showing hierarchical clustering of changed DEGs of rats in CCI group compared with control group. In clustering analysis, upregulated and downregulated genes are colored in red and green, respectively. **c** STRING analysis for biological interactions within DEGs of RNA-seq datasets, involved in immune and inflammatory function. The line color indicates the type of interaction evidence, and line thickness indicates the strength of data support. **d** Quantitative reverse transcription-PCR (qRT-PCR) analysis for differences in expression levels of DEGs in the dorsal horn between CCI models and controls. Results were calculated by normalizing to β-actin in the same sample with the ΔCt method. Changes in relative levels of gene mRNAs expressed as folds of controls. All values were mean ± SEM. *p < 0.05 (*n* = 3)

The DEGs between CCI model and control were subjected to KEGG pathway enrichment analysis using the software KOBAS. The *p* value < 0.05 was set as the threshold of significant enrichment. Based on the KEGG pathway enrichment analysis, the upregulated DEGs were significantly enriched in 14 signaling pathways, such as complement and coagulation cascades, B cell receptor signaling pathway, cytokine-cytokine receptor interaction, and Fc gamma R-mediated phagocytosis signaling pathway, which were mostly related to immune and inflammatory responses (Fig. 4a, Additional file 10: Table S10). However, none of the downregulated gene was significantly enriched in any KEGG pathway.

### Hierarchical cluster analysis of DEGs

To elucidate the role of DEGs in CCI model tissues, DEGs were hierarchically clustered dependent on the gene enrichment features of control against CCI model tissues (Fig. 4b). The most prominently upregulated genes consisted of families of chemokines (Cxcl13 and Ccl2), complement components (C1qc, Ccl2, C1qa, C3, C1qb, and C4a), Fc fragment receptors (Fcer1g, Fcgr3a, Fcgr1a, and Fcgr2b), cluster of differentiations (Cd22, Cd33, Cd53, and Cd68), and G protein-coupled receptors (Gpr31, Gpr34, and Gpr183). Strikingly, chemokine genes showed the greatest upregulation such as Cxcl13 (6.426 fold increase). Most of these genes which have previously been linked to immune and inflammation-related pathways have not been reported in neuropathic pain; and only 20 genes (e.g., Cxcl13, C1qc, Ccl2, C1qa, Fcer1g, Ngfr, Cd53, Cd68, Dusp1) have been demonstrated to be involved in this pathogenesis. This clustering analysis of RNA-seq data will indicate that the DEGs in CCI model are closely associated with the development of neuropathic pain.

### PPI network analysis

To investigate the interaction and hub genes of DEGs involved in pathogenesis of neuropathic pain, the DEGs PPI network were constructed using STRING. The results demonstrated that the predicted PPI in CCI rats were driving the complex interaction network at 14 days after CCI (Fig. 4c). The established PPI network (PPI enrichment *p* value < 1.0e−16) contained 58 nodes (hub genes) and 77 edges (interactions). Five of the top genes with relatively high connectivity degrees (≥ 11) were highlighted: Cd68 (degree = 14), C1qc (degree = 12), C1qa (degree = 11), Laptm5 (degree = 11), and Fcgr3a (degree = 11). Many novel DEGs that we screened may play an important role through regulation of protein expression in neuropathic pain, but future in-depth studies are required.

### Validation by qRT-PCR

To evaluate the reliability of the Illumina sequencing technology, 13 DEGs (11 upregulated and 2 downregulated genes) were randomly selected and detected by qRT-PCR. Figure 4d shows that the upregulation or downregulation trend of candidate genes between CCI-induced model and control group revealed by the qRT-PCR data is congruent with that revealed by RNA-seq method. The result of qRT-PCR analyses provides evidence that the RNA-seq method for the large-scale gene expression quantification was reliable.

## Discussion

In this study, we profiled gene expression in the dorsal horn following CCI-induced neuropathic pain, using RNA-seq method. Sixty-three DEGs were identified in CCI rat model, including 56 upregulated and 7 downregulated genes. We also predicted potential functions of DEGs using GO, KEGG pathway, and PPI network analysis in the CCI model. These findings prompted the proposal that DEGs played a significant role in neuropathic pain processing, and sequencing analysis revealed a potential therapeutic target of neuropathic pain.

Accumulating evidence showed that Cxcl13 and Ccl2 are known to be involved in pathogenesis of neuropathic pain via different forms of neuron-glia interaction in the spinal cord [20, 21]. The chemokines by binding to the G protein-coupled receptors play an essential role in pathological pain conditions triggered by either peripheral inflammation or nerve injury [23, 24]. Our results demonstrated that chemokine genes showed the greatest upregulation, such as Cxcl13 (6.426 fold increase), suggesting that chemokines play a crucial role in the development of neuropathic pain. Furthermore, complements, a key component of the innate immune system and potentially important trigger of some types of neuropathic pain, have been associated with neuroinflammation [22, 25, 26]. Previous study showed that C1qc, C1qa, and Fcer1g might contribute to the generation of neuropathic pain after SNI via immune and defense pathways [26]. Activation of Cd68, the inflammatory microglia-dominant molecule, could result in neuropathic pain in mice with peripheral nerve injury [27]. Cd53, the inflammation-related gene, is chronically upregulated after spinal cord injury [28, 29]. The up-expression of nerve growth factor (NGF) and the NGF receptor is involved in regulating the function of sensory and the development of neuropathic pain [30]. In the present study, upregulated genes included chemokines, complement components, Fc fragment receptors, cluster of differentiations, G protein-coupled receptors, and NGF receptor. Most of the genes are well known in neuropathic pain (e.g., Cxcl13, C1qc, Ccl2) [20–30], suggesting DEGs with the differential functions in diverse cellular pathways, and many are involved in neuropathic pain development and progression.

Previous study demonstrated that Urgcp plays a critical role in glial cell cycle and cell proliferation [31]. Dusp1, a MKP-1, plays a pivotal role in controlling MAPK-dependent inflammatory responses [32]. In the present study, we found that Urgcp and Dusp1 displayed obvious down-expression in CCI rats, which would provide a better understanding of immune and glia cell proliferation, as well as MAPK-dependent inflammatory response abnormalities involved in the neuropathic pain of CCI rats.

In order to obtain insights into DEGs function, GO analysis annotation was applied to the DEG gene pool. GO terms for biological process categories included immune system process, phagocytosis, defense response, and response to external stimulus. Several studies using SNI rat model have reported that key higher expressed genes included those associated with immune and inflammatory pathways in neuropathic pain [20–30], similar to those in CCI model we observed. GO functional analysis showed that downregulated DEGs might associate with inactivation of MAPK activity in CCI models. Inhibition of MAPKs results in downregulation of downstream molecules (cytokines, chemokines, nitric oxide, etc.) in immunocompetent cells and depresses the excitability of neurons in the spinal cord [33, 34]. Therefore, spinal MAPK signaling pathway, such as p38-MAPK (p38) or extracellular receptor-activated kinases (ERKs), may play an important role in the development of chronic allodynia in CCI [35].

PPI network analysis showed that Cd68, C1qc, C1qa, Laptm5, and Fcgr3a were crucial nodes with high connectivity degrees. Laptm5 and Fcgr3a which have previously been linked to immune and inflammation-related pathways have not been reported in neuropathic pain [36, 37]; and other three genes (Cd68, C1qc, and C1qa) have been demonstrated to be involved in this pathogenesis [26, 27]. Previous study showed that Laptm5 is involved in the dynamics of lysosomal membranes associated with microglial activation after nerve injury [36]. Upregulation of Fcgr3a increased the microglial phagocytic capacity in neuroinflammation [37]. It might be inferred that Laptm5 and Fcgr3a are neuroinflammation-related genes that influence neuropathic pain behavior after CCI.

## Conclusions

In conclusion, our results suggest that genes involved in immune and defense responses are affected most significantly after CCI. Genes like Cxcl13, Cd68, C1qc, Laptm5, and Fcgr3a are crucial for neuropathic pain after CCI in rat models. These genes could be used as novel diagnostic and therapeutic targets against CCI-induced neuropathic pain. However, the predicted expressions and interactions need to be further validated by extensive experiments.

## Additional files

Additional file 1: **Table S1.** All expressed transcripts in chronic constriction injury (CCI) model rats. (XLSX 3674 kb)

Additional file 2: **Table S2.** All expressed genes in chronic constriction injury (CCI) model rats. (XLSX 2944 kb)

Additional file 3: **Table S3.** Differentially expressed genes (DEGs) in chronic constriction injury (CCI) model rats. (XLSX 19 kb)

Additional file 4: **Table S4.** Biological processes (BP) result of the upregulated differentially expressed genes (DEGs) in chronic constriction injury (CCI) model rats. (XLS 162 kb)

Additional file 5: **Table S5.** Cellular component (CC) result of the upregulated differentially expressed genes (DEGs) in chronic constriction injury (CCI) model rats. (XLS 34 kb)

Additional file 6: **Table S6.** Molecular function (MF) result of the upregulated differentially expressed genes (DEGs) in chronic constriction injury (CCI) model rats. (XLS 33 kb)

Additional file 7: **Table S7.** Biological processes (BP) result of the downregulated differentially expressed genes (DEGs) in chronic constriction injury (CCI) model rats. (XLS 52 kb)

Additional file 8: **Table S8.** Cellular component (CC) result of the downregulated differentially expressed genes (DEGs) in chronic constriction injury (CCI) model rats. (XLS 28 kb)

Additional file 9: **Table S9.** Molecular function (MF) result of the downregulated differentially expressed genes (DEGs) in chronic constriction injury (CCI) model rats. (XLS 29 kb)

Additional file 10: **Table S10.** Kyoto Encyclopedia of Genes and Genomes (KEGG) result of the upregulated differentially expressed genes (DEGs) in chronic constriction injury (CCI) model rats. (XLS 35 kb)

## Abbreviations

BP: Biological processes
CC: Cellular component
CCI: Chronic constriction injury
DAVID: Database for Annotation, Visualization and Integrated Discovery
DEGs: Differentially expressed genes
ERKs: Extracellular receptor-activated kinases
FPKM: Fragments per kilobase of exon per million fragments mapped
GO: Gene Ontology
KEGG: Kyoto Encyclopedia of Genes and Genomes
KOBAS: KEGG Orthology-Based Annotation System
MAPK: Mitogen-activated protein (MAP) kinases
MF: Molecular function
MKP: MAP kinase phosphatase
MWT: Mechanical withdrawal threshold
NGF: Nerve growth factor
PPI: Protein-protein interaction
qRT-PCR: Quantitative reverse transcription-PCR
RNA-seq: RNA sequencing
SNI: Spared nerve injury
TWL: Thermal withdrawal latency
